# Self-administered versus interview-based questionnaires among patients with intermittent claudication: Do they give different results? A cross-sectional study: Questionários autoadministrados *versus* administrados por entrevistador para pacientes com claudicação intermitente: Os resultados são diferentes? Um estudo transversal

**DOI:** 10.1590/1516-3180.2015.01733009

**Published:** 2016-01-19

**Authors:** Francisco Lozano, José María Lobos, José Ramón March, Eduardo Carrasco, Marcello Barbosa Barros, José Ramón González-Porras

**Affiliations:** I MD, PhD. Angiology and Vascular Surgery Department, Hospital Universitario de Salamanca and Instituto de Investigación Biomédica de Salamanca (IBSAL), Salamanca, Spain.; II MD, PhD. Primary Care Physician, Centro de Salud Villablanca, Madrid, Spain.; III MD. Angiology and Vascular Surgery Department, Hospital Universitario de Getafe, Madrid, Spain.; IV MD. Primary Care Physician, Centro de Salud Jesús H. Gómez Tornero, Abarán, Murcia, Spain.; V MD, PhD. Angiology and Vascular Surgery Department, Hospital Universitario de Valladolid, Valladolid, Spain.; VI MD, PhD. Hematology Department, Hospital Universitario de Salamanca and Instituto de Investigación Biomédica de Salamanca (IBSAL), Salamanca, Spain.

**Keywords:** Intermittent claudication, Quality of life, Questionnaires, Validation studies, Peripheral arterial disease

## Abstract

**CONTEXT AND OBJECTIVE:**

Many clinical investigations use generic and/or specific questionnaires to obtain information about participants and patients. There is disagreement about whether the administration method can affect the results. The aim here was to determine whether, among patients with intermittent claudication (IC), there are differences in the Walking Impairment Questionnaire (WIQ) and European Quality of Life-5 Dimension (EQ-5D) scores with regard to: 1) the questionnaire administration method (self-administration versus face-to-face interview); and 2) the type of interviewer (vascular surgeon, VS, versus general practitioner, GP).

**DESIGN AND SETTING:**

Cross-sectional observational multicenter epidemiological study carried out within the Spanish National Health Service.

**METHODS:**

1,641 evaluable patients with IC firstly completed the WIQ and EQ-5D questionnaires and then were interviewed by their doctor on the same day. Pearson correlations and Chi-square tests were used.

**RESULTS:**

There was a strong correlation (r > 0.800; P < 0.001) between the two methods of administering the WIQ and EQ-5D questionnaires, and between the VS and GP groups. Likewise, there was a high level of concordance (P > 0.05) between the different dimensions of the WIQ-distance and EQ-5D (self-administration versus face-to-face) in the VS and GP groups.

**CONCLUSION:**

There was no difference between the different methods of administering the WIQ and EQ-5D questionnaires, among the patients with IC. Similarly, the two types of interviewers (VS or GP) were equally valid. Therefore, it seems unnecessary to expend effort to administer these questionnaires by interview, in studies on IC.

## RESUMO

**CONTEXTO E OBJETIVO:**

Muitas investigações clínicas usam questionários genéricos e/ou específicos para obter informações sobre os participantes e pacientes. Não se sabe se o modo de administração pode afetar os resultados. O objetivo foi determinar se, nos pacientes com claudicação intermitente (CI), existem diferenças nas pontuações do Walking Impairment Questionnaire (WIQ) e do European Quality of Life-5 Dimension (EQ-5D) no que diz respeito a: 1) a forma de administrar o questionário (autoadministrado *versus* entrevista presencial); e 2) o tipo de entrevistador: cirurgião vascular (CV) ou médico generalista (MG).

**TIPO DE ESTUDIO E LOCAL:**

Estudo epidemiológico observacional, transversal, multicêntrico realizado no Serviço Nacional de Saúde espanhol.

**METODO:**

1.641 pacientes avaliáveis com CI completaram inicialmente o WIQ e questionários EQ-5, e depois, no mesmo dia, foram entrevistados pelo seu médico. Foram utilizados correlações de Pearson e testes de qui-quadrado.

**RESULTADOS:**

Houve forte correlação (r > 0,800; P < 0,001) entre os dois métodos de administração do WIQ e EQ-5D; e entre os grupos CV e MG. Também houve alto nível de concordância (P > 0,05) entre as diferentes dimensões do WIQ-distância e EQ-5D (autoadministrado *versus* entrevista presencial), nos grupos CV e MG.

Conclusão: Em pacientes com CI, não há diferenças entre as diferentes formas de administrar os questionários WIQ e EQ-5D. Da mesma forma, os dois tipos de entrevistador (CV ou MG) foram igualmente válidos. Portanto, não parece necessário despender esforço para administrar esses questionários através de entrevista, em estudos de CI.

## INTRODUCTION

Many epidemiological and clinical studies, including clinical trials, use information directly reported by study participants. There are many generic and disease-specific questionnaires reported in the medical literature that are used by researchers to obtain relevant information about their patients. Research into peripheral arterial disease (PAD) and, specifically, intermittent claudication (IC) also uses generic or specific questionnaires to measure outcomes such as deterioration in walking or health-related quality of life (HRQOL). The former include the Walking Impairment Questionnaire (WIQ), while in the European context, the European Quality of Life-5 Dimensions (EQ-5D) is recommended for evaluating HRQOL among patients with IC.^1^^,^^2^

Most of these questionnaires have been validated from the original version to other languages; for example, the WIQ has been validated in Portuguese and Spanish.^3^^,^^4^^,^^5^ The WIQ and EQ-5D were originally designed to be self-administered and have been used in that way in most studies on IC.^6^^,^^7^ However, these questionnaires may be completed by other means; for example, with the aid of an interviewer (face-to-face or by telephone). While the latter methods have the advantage of directly controlling the process and thereby offer the possibility of obtaining superior-quality results, self-administration methods (in the consultation room or at home, and returned by post or e-mail) do not require research staff, enable lower research costs and allow patients greater freedom to express their responses to the questions. However, only Conley et al. have evaluated the effects of the two methods of administering the WIQ.^8^ Thus, while some studies (not on IC) have shown differences between administration formats,^9^^,^^10^^,^^11^^,^^12^ others have found no statistically significant differences.^13^^,^^14^^,^^15^^,^^16^^,^^17^ For these reasons, in some epidemiological studies, self-administered and interviewer-led questionnaires are available to accommodate the preferences, physical impediments or literacy of the participants.

## OBJECTIVE

Our study aimed to examine whether, in a large cohort of patients with IC who had completed the WIQ and EQ-5D questionnaires, there were systematic differences in the scores that could be attributed to: 1) the administration method of the questionnaire (self-administration versus interview-based); and 2) the type of interviewer (vascular surgeon versus general practitioner).

## METHODS

A cross-sectional observational multicenter epidemiological study on IC in Spain was carried out between May and December 2011, using previously published data.^18^

Vascular surgeons (VSs) and general practitioners (GPs) were identified through the scientific societies participating in the study. All the physicians had previously taken part in epidemiological studies about some type of vascular pathology. Patients were recruited during visits to hospitals (in the case of the VSs) or health centers (in the case of the GPs) within the National Health Service. Each researcher was obliged to include 3-4 consecutive patients affected by IC. The diagnosis of IC was made through the clinical history (including a positive Edinburgh questionnaire result), physical examination and ankle-brachial index (ABI < 0.90 or > 1.3, in diabetic patients), following previously described methods.^19^^,^^20^ The ABI of each extremity was calculated by dividing the highest pressure obtained in either of the leg arteries by the maximum brachial value. In the records of each patient, only the claudicant limb, or the lower ABI in the bilateral cases, was taken into account.^21^

Each physician compiled the information on a data collection sheet covering the demographic and clinical characteristics of the patient. Two questionnaires were completed on the same occasion: WIQ and EQ-5D.

The WIQ is a questionnaire specific to IC that evaluates four parameters: pain, distance covered, speed and stair-climbing ability.^6^ For each domain, a score for the ranked difficulty of doing each of the items is calculated, ranging from 0 (total incapacity) to 100 (full capacity). The Spanish version of the questionnaire was used.^5^

The EQ-5D questionnaire is an instrument designed by a European group for measuring HRQOL.^7^ It is a generic questionnaire that is widely used in research because of its ease of use. It has three components. The first evaluates five factors: mobility, self-care, everyday activities, pain/discomfort and anxiety/depression. The scores obtained are summarized as an overall index from 0 (worst state of health) to 1 (best state of health). The second part consists of a visual analogue scale (VAS), in the form of a thermometer, with end points of “worst” and “best” imaginable state of health (scored from 0 to 100, respectively). The third part (patient preference values) was not evaluated. The Spanish version, which has also been validated for use in primary care, was used.^22^^,^^23^

Both questionnaires were administered through two methods: self-administration (before the start of the aforementioned consultation), and a subsequent face-to-face interview by the physician (VS or GP). We applied the questionnaire in these two forms without any relevant time interval between them. Initially, the patients completed the WIQ and EQ-5D, in that order, without any help. Once that phase was completed, they filled in the questionnaires in the same order with the help of the doctor. We can consider the latter phase to be the ‘ideal means of applying the questionnaires’ or to be a control for the former phase. The formats of the self-administered and interview-based questionnaires were identical.

### Statistical analysis

A data file was created in PASW (version 18; IBM, New York, NY, USA). Continuous variables were summarized as means and standard deviations; categorical variables were expressed as percentages. Normally and non-normally distributed continuous variables were compared using Student’s t test and the Mann-Whitney U test, respectively. Categorical variables were examined using the χ^2^ or Fisher’s exact test; the latter was used when the expected frequencies of one or more categories was less than 5. The Pearson correlation coefficient (r) of each dimension of the WIQ and EQ-5D was calculated for each of the two methods of questionnaire administration. Values of r were interpreted as follows: 0.0-0.19, very weak correlation; 0.20-0.39, weak; 0.40-0.59, moderate; 0.60-0.79, strong; and 0.80-1.0, very strong.^24^ All comparisons were based on a 95% confidence interval. Results were taken to be statistical significant when P < 0.05.

### Ethics approval for the study

The study was approved by the Scientific and Ethics Committees of the Hospital Clinic (Barcelona, Spain) on March 10, 2011 (Protocol: SEA-NUL_2011_01).

## RESULTS

Six hundred and twenty-five researchers provided information from 2,127 consecutive patients affected with IC, of whom 486 patients (22.8%) were removed from the study because their data were incomplete. Thus, 1,641 patients were evaluated: 920 patients from 249 VSs and 721 from 247 GPs. The reasons for exclusion and removal are shown in Table 1. Patients and researchers were selected from across the entire country, including urban and rural areas.

**Table 1.**
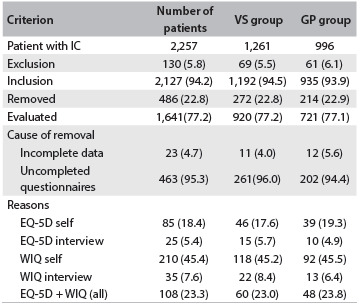
Patients with intermittent claudication (IC) and criteria for inclusion, exclusion and withdrawal*

*Number (percentage). EQ-5D = European Quality of Life-5 Dimension; WIQ = Walking Impairment Questionnaire.

The demographic and clinical characteristics of the groups are summarized in Table 2. Patients in the VS group had lower ABI than those in the GP group (0.63 versus 0.71; P < 0.001).

**Table 2.**
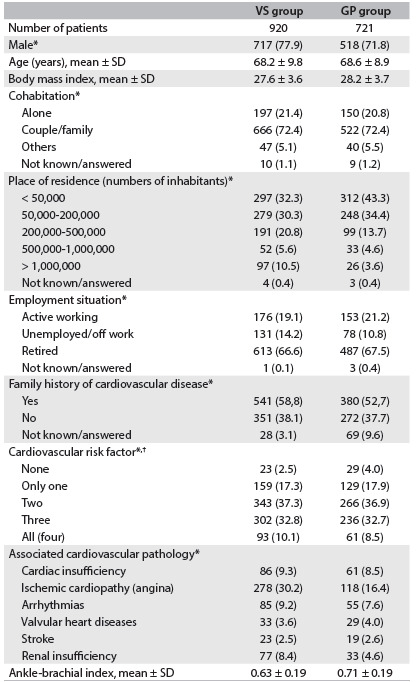
Baseline characteristics of patients with intermittent claudication

*Number (percentage) or mean ± standard deviation (SD). VS = vascular surgeon; GP = general practitioner; ^†^(tobacco, diabetes mellitus, arterial hypertension and dyslipidemia).

The scores for the various components of the WIQ, self-administered and administered through an interview with a VS or GP, are shown in Table 3. The group of patients interviewed by the VSs had significantly worse scores for the dimensions of pain (47.1% versus 50.4%; P < 0.001) and distance covered (34.1% versus 36.4%; P = 0.007).

**Table 3.**
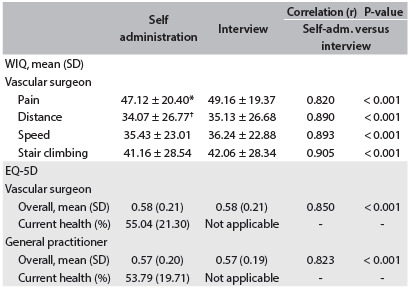
Walking Impairment Questionnaire (WIQ) and EuroQol (EQ-5D). Survey mode

*P < 0.001, between pain assessed by vascular surgeon and pain assessed by general practitioner; ^†^P = 0.007, between distance assessed by vascular surgeon and distance assessed by general practitioner. SD = standard deviation.

The WIQ scores obtained from both types of interviewer were somewhat higher on average than those obtained through self-reporting. However, only the higher score in the VS group (mean score 2.04 points higher) was statistically significant (P < 0.05).

The correlations between self-administration and interview-based methods of completing the questionnaire were very strong (r = 0.820-0.905), even when considering the two types of interviewer separately (Table 3).

The overall scores from the EQ-5D questionnaire were almost identical (0.58 ± 0.21) when administered through self-reporting and through interview with a VS, and likewise when the interviewer was a GP (0.57 ± 0.21). There were no differences between the VS and GP groups (P = 0.429). The correlation between the two methods of administering the questionnaire was very strong, whether the interviewer was a VS (r = 0.850; P < 0.001) or a GP (r = 0.828; P < 0.001).

Tables 4 and 5 compare the results from the various dimensions of the WIQ and EQ-5D obtained through self-administration and through interviews conducted by a VS or GP. No differences were found between these methods (P > 0.05).

**Table 4.**
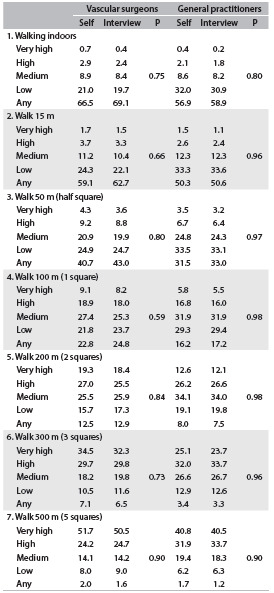
Walking Impairment Questionnaire: distance dimensions (%) through self-administration or interview, by vascular surgeons and general practitioners

Self = self-administration.

**Table 5.**
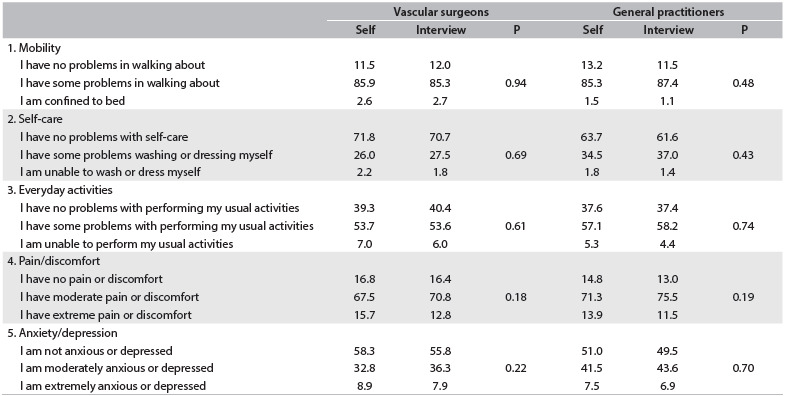
EuroQol (EQ-5D) dimensions (%) through self-administration or interview, between vascular surgeons and general practitioners

Self = self-administration.

## DISCUSSION

Our results demonstrate that self-administration and interview by a physician are both valid approaches for evaluating patients with IC using the WIQ and EQ-5D questionnaires. Likewise, the correlation between the two modes of administering the questionnaires was high for both types of interviewer (VS and GP).

These findings are highly important, since if the different ways of administering a particular questionnaire were to produce different results in an epidemiological study or clinical trial, the estimates of the associations or the effects of the treatment investigated could be affected, thus raising the possibility of incorrect conclusions being drawn.

It is possible to measure an individual’s capacity to walk. The treadmill widely used by VSs enables objective measurement of the walk of patients with IC and evaluation of the changes following an intervention program. Introduction of the WIQ has helped GPs measure the walking capacity of their patients without the need for sophisticated equipment. The WIQ has become a widely used instrument for evaluating the walking capacity of patients with claudication,^25^ and its scores correlate well with more objective measurements.^26^^,^^27^ The WIQ has been used jointly with various HRQOL questionnaires, thereby providing excellent overall information about the limitations of these patients.

IC patients have significantly poorer HRQOL than healthy controls, especially with regard to the physical domains. Of the various questionnaires that measure HRQOL, the EQ-5D has the great advantages of being simple to use (only five dimensions) and of being validated for IC, despite its generic nature.^28^^,^^29^

As Puhan et al. noted,^15^ most questionnaires of this type tend to be self-completed. Indeed, the WIQ and EQ-5D were originally designed to be self-administered, although they may also be completed through personal interview, over the telephone or by post. The aforementioned advantages and disadvantages make it necessary to compare these methods.

With regard to the WIQ, we are aware of only one study, by Coyne et al.,^8^ that has compared the different administration methods. Although their study included a relatively small sample of 60 patients, it sought to validate the questionnaire for self-administration and telephone interviews, and found no significant differences in the scores on the four subscales of the WIQ (pain severity, distance, speed and stair-climbing), compared with control interviews. Our study also found no differences in the results obtained through the various forms of administration. In this type of study, in which a degree of psychological influence may be expected, a correlation coefficient of 0.70 or more can be considered to be very strong.^30^

Nevertheless, there were two notable observations:

1. As in other studies, albeit on other pathological conditions,^31^ we noted numerically higher scores when the questionnaires were administered by an interviewer, although the difference was only significant for the pain domain of the WIQ. This is commonly explained as being the result of social desirability bias,^32^ in which participants may state that they are less affected when interviewed by research staff than when the questionnaires are self-administered.

2. As in the study by Mahe et al.,^33^ there was also a substantial number of errors and missing responses in the self-administered WIQ questionnaires. These patients were excluded from the study. In order to avoid any bias that may arise as a consequence, completion of the questionnaire would have to be supervised. In our study, the excluded and included patients did not differ with regard to items that might influence the socioeconomic characteristics of the two groups: age, sex, cohabitation, place of residence and type of job.

Although the EQ-5D has been widely used in cardiovascular studies,^34^^,^^35^ there are no published studies comparing the different formats for administering this questionnaire to patients with this type of pathological condition. The best match for the EQ-5D has been found among HIV patients, whose scores were very similar for the different administration methods (self-reporting versus interview) and types of interview (face-to-face versus telephone).^13^

Our study has some limitations. Apart from the considerable number of excluded patients, the study was also limited because it was not possible to carry out treadmill tests to measure the IC objectively. The use of ergometers is time-consuming and costly and requires control by specialist professionals. However, this omission may not be too important, since clinical manifestations and the WIQ can be used as an alternative to treadmill testing for objectively assessing functional walking ability. Forty percent of the patients recruited came from non-hospital consultations, thus justifying the use of the WIQ.

Another limitation of our study was the absence of a washout period between the two forms of administration of the questionnaires. In our study, this was sequential: first, self-administration (trial form); second, face-to-face (ideal application or control). Our aim was not to evaluate either of the questionnaires but to measure the equivalence between the two forms of administration. Evidently, when patients arrived at the interview they already had previous (and recent) experience, but this was minimized due to the doctor’s influence on filling out the questionnaires. On the other hand, the doctor would have a decisive influence, were the sequence to be interview first, followed by self-administration.

## CONCLUSION

Our study, which was carried out on a large sample of patients with intermittent claudication, provides evidence that the format of administration of the WIQ and EQ-5D questionnaires has no significant effect on the measurements, provided that the patient is able to fill out a self-applicable form. Consequently, it is not necessary to take into consideration the different formats of administration in this type of study, in analyzing the results within this scenario. This is fortuitous in that it avoids having to do unnecessary work. However, researchers should carefully consider the format of administration used, in order to avoid bias arising from the application method of the questionnaire chosen.
